# Primary Health Care and Disasters: Applying a “Whole-of-Health System” Approach through Reverse Triage in Mass-Casualty Management

**DOI:** 10.1017/S1049023X23006246

**Published:** 2023-10

**Authors:** Andrea Alesi, Michelangelo Bortolin, Luca Ragazzoni, Alessandro Lamberti-Castronuovo

**Affiliations:** 1. CRIMEDIM—Center for Research and Training in Disaster Medicine, Humanitarian Aid and Global Health, Università del Piemonte Orientale, 28100 Novara, Italy; 2. Department for Sustainable Development and Ecological Transition, Università del Piemonte Orientale, 13100 Vercelli, Italy

**Keywords:** H-EDRM, mass casualty, primary health care, reverse triage, whole-of-health system

## Abstract

**Introduction:**

In 2019, the World Health Organization (WHO) published the Health Emergency and Disaster Risk Management (H-EDRM) framework detailing how effective management of disasters, including mass-casualty incidents (MCIs), can be achieved through a whole-of-health system approach where each level of the health care system is involved in all phases of the disaster cycle. In light of this, a primary health care (PHC) approach can contribute to reducing negative health outcomes of disasters, since it encompasses the critical roles that primary care services can play during crises. Hospitals can divert non-severe MCI victims to primary care services by applying reverse triage (RT), thereby preventing hospital overloading and ensuring continuity of care for those who do not require hospital services during the incident.

**Study Objective:**

This study explores the topic by reviewing the literature published on early discharge of MCI victims through RT criteria and existing referral pathways to primary care services.

**Methods:**

A scoping literature review was performed and a total of ten studies were analyzed.

**Results:**

The results showed that integrating primary care facilities into disaster management (DM) through the use of RT may be an effective strategy to create surge during MCIs, provided that clear referral protocols exist between hospitals and primary care services to ensure continuity of care. Furthermore, adequate training should be provided to primary care professionals to be prepared and be able to provide quality care to MCI victims.

**Conclusion:**

The results of this current review can serve as groundwork upon which to design further research studies or to help devise strategies and policies for the integration of PHC in MCI management.

## Introduction

In recent years, a new comprehensive approach to disaster management (DM) has been developed, in which coordination of every level of the health system is encouraged.^1^ Supporting this approach, in 2019, the World Health Organization (WHO; Geneva, Switzerland) published the Health Emergency and Disaster Risk Management (H-EDRM) framework which details the contributions of all health system actors in reducing risks and consequences of disasters and mass-casualty incidents (MCIs).^2^ The H-EDRM’s whole-of-health system approach emphasizes the need for multisectoral policies and a practical integration of all health services in DM. Consequently, a primary health care (PHC) approach can be key in reducing disasters’ negative health outcomes, since it encompasses the critical roles that primary care services play during crises along with multisectoral policies and empowered communities.^3,4^ When effectively integrated in DM plans, primary care facilities help prevent hospitals overloading by providing on-going treatment to less severe victims, maintaining continuity of care and effective risk communication for those who do not require hospital services during the incident.^5^ During an MCI, the local health care system is overwhelmed if the number of casualties exceeds resources during a short period of time.^6^ To increase surge capacity, health care workers (HCWs) can determine which patients can safely be discharged through a reverse triage (RT) strategy. Through RT, patients at low risk of adverse events, who do not require major medical assistance for at least 96 hours, can be discharged home or to other primary care facilities (eg, nursing homes).^7–9^ Nevertheless, primary care professionals generally feel unprepared to engage in MCIs because they are often excluded from DM policies and training opportunities.^10,11^ Countries’ DM plans and MCI management have historically focused on hospitals and few policies exist for how to practically integrate such primary care facilities in crisis response.^5^ Research in this area is encouraged, especially after the coronavirus disease 2019 (COVID-19) pandemic has exposed the inadequacy of health systems leading to a fragmented management of the emergency.^12^ Implementing a RT strategy that engages primary care directly is the logical first step towards integrating PHC into DM. The goal of this paper is to explore the extent of the current literature addressing the integration of a PHC approach in MCI response as a way to increase in-hospital surge capacity and improve access to care. Through a systematic review of the literature, this study collects evidence around existing RT criteria used to discharge patients to primary care (ie, home or nursing homes) alongside existing referral pathways from hospitals to primary care in an MCI response. The results will hopefully contribute to building knowledge around the nature of the integration of PHC into DM as a way to increase in-hospital surge capacity. It can serve as groundwork upon which to design studies or policies for the integration of the PHC approach in MCI management.

## Methods

This scoping review was conducted in February 2023 following the Preferred Reporting Items for Systematic Reviews and Meta-Analyses (PRISMA) checklist (Supplementary Material, File S3; available online only). The search was performed using three databases: PubMed (National Center for Biotechnology Information, National Institutes of Health; Bethesda, Maryland USA), Web of Science (Clarivate Analytics; London, United Kingdom), and Scopus (Elsevier; Amsterdam, Netherlands). The search string was created with keywords related to MCI management and PHC using Boolean operators (Supplementary Material, File S2; available online only). No time limit was applied to the research. The Rayyan tool^13^ was used to delete duplicates and perform the selection process. The screening of titles and abstracts was performed by AA, ALC, and MB against the agreed criteria. The selected articles were original studies: (1) describing a hospital response to sudden patient influx after an MCI; (2) considering early patients discharge to primary care (ie, home or nursing homes); and (3) written in English. The reference lists of all relevant articles were screened for additional studies. Articles were excluded when they did not address MCIs, or whenever RT was used to discharge patients within the hospital (eg, step down from intensive care units [ICUs]). An extraction sheet (Supplementary Material, File S3; available online only) was developed a priori and used to extract relevant information. This helped categorize information about the study, including the criteria used to discharge patients from hospitals after an MCI, the surge capacity that ensued, and any extant barriers and facilitators to discharging patients to primary care early after MCIs. AA and ALC independently extracted information from the studies and categorized them. The categorization was discussed and consolidated upon reaching agreement within the research team.

## Results

**Figure 1.**
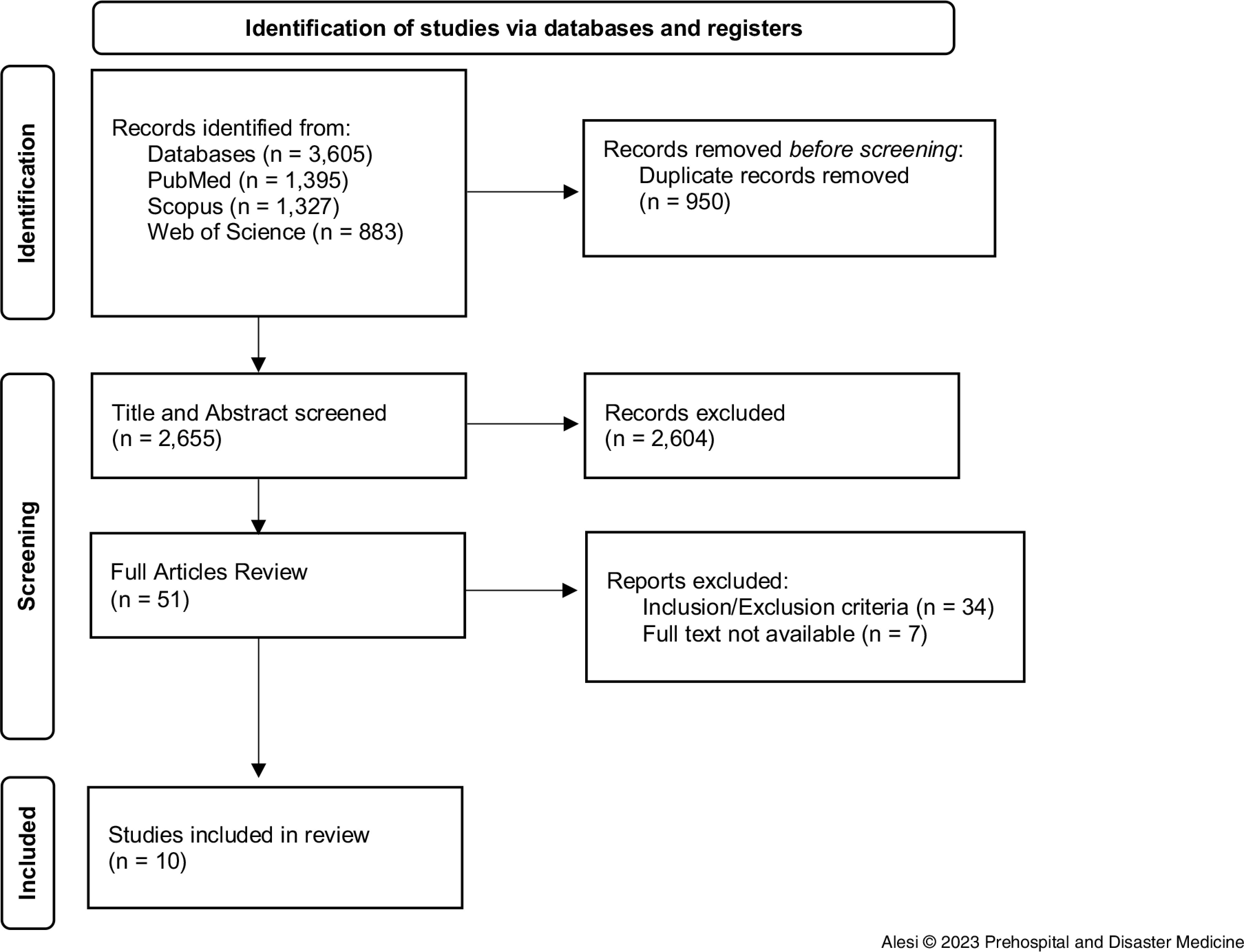
PRISMA Checklist for the Identification of Studies.

A total of 3,605 records were identified from the databases. Duplicates were removed, leaving 2,655 articles to be judged for relevance. Of those, 2,604 were excluded by title and abstract, leaving 51 records whose full text was screened. After evaluation, ten studies were included in this review (Figure 1).

Most of the articles described a simulated MCI scenario without specifying the type, except one describing a gas attack.^14–22^ Satterthwaite (2012) was the only one describing the response to a real event. Seven out of ten studies involved one hospital.^16,18–23^ Two studies involved, respectively, three and four hospitals within the same region.^14,15^ One involved all acute hospitals in New York City (New York USA).^17^ Three studies targeted pediatric populations.^16,18,20^ One study included only adults,^15^ while another included both adult and pediatric in-patients.^22^ Almost all studies aimed at investigating RT efficacy in creating hospital surge capacity by discharging patients to primary care early in an MCI response.^14–21,23^ Among them, one investigated what resources were needed at the primary care level.^19^ Finally, the purpose of the study by Pollaris (2018) was to test a tool to guide clinical decision making in the RT process.

### Criteria Used for RT

The studies’ patients were discharged based on the following criteria: (1) by assessing patients’ risk of facing a consequential medical event (CME) after early hospital discharge;^15,16,22^ (2) by using discharge tools addressing barriers to discharge home;^17,18^ (3) through HCW clinical evaluation;^20,21,23^ and (4) by assessing the potential patient disposition based on the level of care required at a variable number of hours after MCIs.^14,19^

In the first group, Kelen developed a classification system based on the patients’ likelihood of developing a CME (ie, unexpected death or irreversible impairment within 72 hours from discharge) for which an in-hospital critical intervention (CI; eg, defibrillation or airway management) would be necessary to stabilize patient conditions. Patients without a CI during the four-day observation period were considered suitable for early discharge. The risk categories and the CI list were developed for adults and children.^15,16^ Pollaris (2017) developed the Reverse Triage Tool Leuven based on Kelen’s version. In the second group, the Rapid Patient Discharge Assessment and the Emergency Discharge Assessment tools considered barriers to discharge home and the need for support for early discharge.^17,18^ The first tool considers both clinical barriers (eg, waiting for lab results) and non-clinical barriers (eg, staff shortage or patient awaiting transportation or refusing to leave). The second was developed for pediatric patients to assess barriers to discharge (eg, social needs, need for intravenous medications, or need of respiratory support). In the third group, the discharge criteria were freely based on clinicians’ assessment.^20,21,23^ Among the included criteria, Bird (2020) chose individuals awaiting in-patient tests that could be performed as out-patients, those whose elective procedures were cancelled, and those patients no longer requiring intravenous medications. Esmalian (2018) based the decisions to discharge patients on hospital protocols concerning the ten most common conditions leading to hospitalization in each ward. In the fourth group, patients were discharged earlier after an assessment of the potential disposition at specific points in time after the MCI. Davis (2005) based the assessment of the potential disposition on the required level of care (eg, more intensive level of care or possible discharge to in-hospital temporary facilities or home). Challen (2006) categorized patients’ disposition based on their demographics and clinical factors, including recent surgery or the need for intravenous therapy.

### Surge Capacity and Adverse Events

The estimated increases in surge capacity that RT yielded in the reviewed papers varied significantly. This was because each paper examined a different health care system and used a different set of discharge criteria. By applying his newly developed classification system, Kelen (2009) prospectively studied elective wards of three capacity-constrained hospitals. The results showed that the percentage of patients who could be discharged early in an academic hospital was 40%, 47% in a teaching facility, and 59% in a community hospital. Slightly lower results were yielded by Davis and Challen.^14,19^ In Jacobs-Wingo’s study (2018), one-fifth of the beds were cleared within the first 48 hours. Esmailian (2018) and Satterthwaite (2018) discovered that 20% of cases were discharged from an Iranian tertiary hospital through RT. Satterthwaite (2018) found that 19 patients (6%) could be sent home at least one day before the expected discharge.

Some studies defined the contribution of each hospital department to surge capacity.^14–16,21^ In Kelen’s paper (2017) targeting the pediatric population, the psychiatry unit had the most patients eligible for immediate RT, accounting for more than one-half of the RT effect. Oncology and ICU had the smallest effect. There was consensus that psychiatry,^15,16^ general surgery, and obstetrics create more surge capacity than others.^14,15,21^ In medical wards, in-patients awaiting investigations that could be carried out as out-patients or patients on intravenous medications who could be switched to oral could create surge capacity.^15,19^

Additional strategies to boost surge capacity were described:^15,16,21,23^ cancellation of elective activities/admissions and/or opening of staffed/unstaffed beds. In Kelen’s paper targeting pediatric populations,^16^ gross surge capacity increased from 10.8% to 57.7% within the first 24 hours thanks to a multipronged approach. Similarly, Kelen reported that early safe discharge of adult in-patients with the aid of pre-determined criteria together with additional strategies could free up to 84% of the beds within four days after the incident.^15^

Pollaris (2018) suggested that having pre-determined criteria prior to MCI response may help reduce the patient population that needed to be evaluated for early discharge to one-third and it doubled the probability of selecting an actual dischargeable patient. Esmailian and Kelen stressed the importance of classification systems for early discharge, especially for pediatric populations and for those hospitals working with high bed occupancy rates that need to increase hospital capacity during MCIs.^16,21^

Three studies reported RT-related adverse events rate in early discharged patients.^15,20,23^ In Kelen’s study,^15^ eight percent of patients eligible for early discharge underwent at least one CI during their in-patient stay. Given that in the majority of cases (74%), the CI was initiated at least six days after T0, the authors argued that the majority of them would still be safe for 96 hours at home. Bird (2020) analyzed the performance of 29 early discharged patients and found that 58% were discharged home within 48 hours. Of those still hospitalized after seven days, only three were considered as inappropriate discharges. In the only real case scenario, seven out of 19 victims were later re-admitted, but only one due to MCI consequences.

### Involvement of Primary Care in MCI Management

Four articles explicitly mentioned discharging patients to low-acuity nursing facilities as a way to increase bed availability while at the same time providing on-going follow up to low-intensity patients.^14,17,18,23^ Seven articles reported discharging patients home.^15–20,23^ However, no article made explicit mention of referral pathways existing across the primary-hospital care continuum to grant continuity of care for the patients that were discharged home.

Transfer-of-care protocols should contain criteria for early discharge and regulate a systematic process of referral of patients from hospitals to primary care with clear handovers.^16,18,20^ The discharging team and modalities need to be made explicit. Discharge plans need to be clear and patients should receive adequate instruction on medications or devices. Hospital protocols should incorporate solutions to frequently occurring barriers to discharge. Challen (2006) reported that, for patients who could be discharged home, hospitals took more than four hours to organize a patient’s transfer due to common barriers to discharge. These barriers were ubiquitous among discharge candidates and frequently revolved around the incompleteness of the patient’s charts,^17^ including missing discharge order, prescription for aftercare, and need for instructions on medications or devices. The availability of adequate workforce and the lack of transportation may represent other barriers to discharge.^14,16–18^ Patient-specific social factors (eg, unavailable clothing, inability to engage in daily activities, or no family/friends’ support) should be considered. Jacobs-Wingo recommended a bed management committee to ensure that discharge procedures were applied quickly,^17^ liaising with all parties (eg, transportation and PHC professionals). Bird (2020) recommended using handwritten summaries over electronic ones and dividing HCWs in admission/discharge teams. Some authors reported the option of having on-site low-intensity facilities directly within a hospital premises in non-traditional patient care areas (eg, parking lots or cafeteria).^16,19^ During MCIs, these facilities may be staffed with low-skilled cadres and have the advantages of rapid access and the proximity to more experienced personnel, thereby simplifying patients’ transfer.

Having standardized discharge criteria is helpful at the primary care level since it may help anticipate the volume of surge capacity to be expected during the response and what resources may be needed at this level.^14,21^ Equipping the primary care system with specific services or boosting HCWs’ competencies according to patients’ needs is key and may account for an extra 13% of the total bed capacity per day.^18^ The most frequent MCI victims’ PHC needs are generally the need for intravenous medications, wound dressings, or a close follow-up. To help ensure the preparedness of such services, Jacobs-Wingo^17^ mentioned a hospital-to-nursing-home decompression project to assess the receiving capacity of long-term care facilities located outside of city-defined evacuation zones during an MCI.

## Discussion

The aim of this study is to explore the literature on the integration of PHC in MCI response through RT as a way to increase hospital surge capacity and improve access to care. The results confirm that RT can be an effective, safe strategy to increase bed availability after an MCI, augmenting surge capacity while conserving resources. It should be included in every hospital disaster contingency plan, regardless of hospital size, level of care, or services provided. When considering which patients can be discharged home, having clear criteria helps making the process safer and more effective. Clinical assessment is irreplaceable when deciding which patients can be discharged home. However, it is also important that hospitals have a classification system based on the level of care required or on the patients’ likelihood of developing a complication if a CI is not performed. Furthermore, patient-specific barriers to discharge home, such as the presence of a discharge plan or the availability of clothing or transportation, need to be considered. Every hospital should plan for RT and perform the necessary studies to validate the appropriate context-specific strategies to increase in-hospital capacity during an MCI.

In the reviewed articles, little information can be found on the practical integration of PHC into MCI management. What research does exist centers the perspective of hospital staff doing the RT itself, with very little emphasis on which measures need to be implemented in primary care in order to grant continuity of care once the patients are discharged. In line with the H-EDRM precepts and as stated in a recent review highlighting the characteristics that render PHC prepared for disasters,^24^ this integration can only take place with clear policy guidelines that coordinate action across the institutions. In MCI response, clear referral protocols need to be developed to ensure that patients’ conditions at home are proactively monitored. Different cadres could be involved in the continuum of care after hospital discharge to grant a PHC system-wide program of care for the victims. Primary care professionals, pharmacists, among others, could play a part in ensuring that victims receive adequate care. The main role can be naturally covered by general practitioners who can coordinate actions across other providers. Adequate training should be provided to them to be able to provide quality care to MCI victims. Involving them directly in MCI simulations may be beneficial, since professionals who attend DM training are generally more willing to take part in emergency response.^25^ Training should tackle general principles of DM (eg, office preparedness) and additional skills (eg, management of disaster-specific conditions including mental health). Attention should be placed to surge capacity at the primary care level itself, since large volumes of patients discharged during MCIs could increase the workload for all PHC actors. Having clear referral protocols between hospitals and primary care networks along with boosting HCWs’ preparedness may result in a greater surge capacity,^26,27^ since hospital professionals would be more prone to engage in early discharge and PHC professionals would feel involved.

The option of low-acuity nursing facilities was mentioned in some studies and should also be considered in MCI management as a critical component of DM. Hospitalized patients could be transferred to “step-down” facilities supervised by primary care professionals and staffed with paramedics. This could contribute to expanding in-hospital surge capacity as the discharge of patients to these facilities is safer, even more so when these facilities are built as temporary structures close to hospitals or on the same premises.^28,29^ During the COVID-19 pandemic, this strategy was successfully implemented to create surge capacity in overwhelmed hospitals.^30,31^

## Limitations

Firstly, the data search did not cover gray literature, hence anecdotal experiences and reports might have been missed. Secondly, all but one study is based on simulated events and results could be different once more evidence from real events is collected. Lastly, a critical appraisal of the retrieved articles was not performed due to the heterogeneity of the studies and because the original purpose of the paper was to map existing evidence on the topic.

## Conclusion

The results of this review show that integrating primary care facilities into DM through the use of RT may be an effective strategy to create surge during MCIs, provided that clear referral protocols and context-specific RT criteria are implemented between hospitals and primary care services to ensure continuity of care. Broadening DM plans to include RT to the primary care system appears to be an effective strategy in MCI response for creating surge capacity, but research on this topic is still in its infancy. Further surge capacity research might benefit from further prospective studies, as recently stated by a recent review on surge research.^8^ In particular, more studies targeting RT toward lower levels of care ought to be made public, particularly those focusing on the involvement of PHC actors.

## Supplementary material

For supplementary material accompanying this paper visit https://doi.org/10.1017/S1049023X23006246. click here to view supplementary material
