# Supplementary material for: Primary Health Care and Disasters: Applying a “Whole-of-Health System” Approach through Reverse Triage in Mass-Casualty Management
Source: Prehosp Disaster Med. 2023 Sep 1;38(5):654–9. doi: 10.1017/S1049023X23006246 (PMC10548020; doi:10.1017/S1049023X23006246)
Supplement: Supplementary file 1 [file S1049023X23006246sup.zip › S1049023X23006246sup001.docx]

Supplementary file S1

| **First Author** | **Year** | **Type of publication** | **Study design** | **Study Methodology** | **Country** | **Type of MCI** | **Type of Hospital** | **Population** | **N. of Patients** | **Criteria** | **Decision Maker** | **Criteria Methodology** | **Surge capacity** | **Adverse Events** | **Referral Pathways** | **Barriers and Facilitators** | **Recommendations** |
| --- | --- | --- | --- | --- | --- | --- | --- | --- | --- | --- | --- | --- | --- | --- | --- | --- | --- |
| Davis DP, et al.^14^ | 2005 | Original Study | Prospective, Cross sectional | Survey | US | Simulated; not specified | N=4 Hospitals;  N=2 level II trauma center (372 and 280 beds)  N=1 Level I trauma center (360 beds)  N=1 Non trauma Hospital (120 beds) | Admitted patients | N=788 | - Age, gender, and admission diagnosis  -Clinical Assessment of the potential disposition in the event of a MCI based on the level of intensive care required (intensive care, step down, nursing facility, home) at 2,24,72h | Nurse Managers; Physicians (the two categories made different disposition assessments) | - | Discharge home:Approximately one-third of all patients at 24 hours and almost half at 72 hours Transferable to a, on-site nursing facility: Approximately one-quarter of the patients at both time points | - | On site Nursing Facility, no referral pathway when discharged home | (I) Site for an hypothetical Nurse Facility (II) Availability of healthcare workers employable in the nurse facility; (III) RT decision Maker: The physicians assessed more patients as either transferable to an on-site nursing facility or dischargeable to home than did the nurse managers, but were less aggressive in transferring or discharging ICU patients when compared to nurses; (IV) Patients’ Clinical Profile: Female gender, Obstetrics, Surgical and Burn patients are associated with higher discharge rates (V)Tertiary vs lower level Hospital | (I) on-site nursing facility using non-traditional patient care areas (II) mathematical modelling of hospital bed surge capacity that predicts dischargeability of patients according to similar diagnostic categories |
| Challen K, et al.^19^ | 2006 | Original Study | Cross-sectional | Survey | UK | Simulated; not specified | Tertiary Hospital  (855 beds) | Admitted patients | - | Study group categorized patients' disposition based on patients' nursing notes (patient demographics + factors such as recent surgery, ongoing intravenous therapy, etc..) and according to the the European amendment of the Appropriateness Evaluation Protocol Primary endpoints:  (I) an immediately available bed (II) discharge feasible within 4 hours, (III) discharge feasible within 4–12 hours, (IV) patient still requiring inpatient care | - | - | 302 beds  (36% of hospital capacity) within 12 hours of an incident, of whom 90 required residential care and 60 community support.  Primary care covering a population of 150k can accomodate around 50–60 patients (25 in patients' homes, 10 in nursing care and 20 in therapy-supported intermediate beds) as surge capacity in the event of a major incident | - | Dischargeable to residential care or to community support. | Social factors might be an obstacle for discharge | Define the potential capacity of PHC and scope the requirement  for its support pre-incident. |
| Kelen GD, et al.^15^ | 2009 | Original Study | Prospective Randomised | Survey | US | Simulated; not specified | N=3 Hospitals;  University Hospital (1017 beds); Teaching affiliate Hospital (355); Community Hospital (260 beds) | Admitted Patients; ICU and Pediatric excluded | N=3491 | 5 risk categories of a consequential medical event (defined as: unexpected death, irreversible impairment, or reduction in function) related to early hospital discharge. The events were considered consequential only if there is an in-hospital Critical Intervention (CI) with the potential to reverse or prevent additional deterioration. Patients without a CI during the 4-day observation period were considered suitable for early discharge. |  | Kelen GD, et al.(2006)  Warfare Analysis Laboratory combined with a consensus process. | Reverse triage made up the majority (50%, 55%, 59%) of surge beds. Most extra-capacity was available within 24 to 48 hours. | 8% of patient suitable for early discharge required a Critical Intervention beyond 96 hours | N=1542 patients discharged to home (44%); N=208 discharged with needs (6%) | (I) The likelihood of not requiring a CI or being discharged during the evaluation period varied somewhat by patients’ primary condition: 61% psychiatric, 61% oncological, 71% surgical, 76% medical, and 93% obstetric.  (II) academic vs community hospital | (I)A larger percentage of patients could be sent home safely or avoid admission should the concept of “hospital at home” be available during a disaster.  (II) community resources availability |
| Satterthwaite P, et al.^23^ | 2012 | Original Study | Case Report | Analysis of Medical  Records review | Australia | Boat explosion | General Hospital (353 Beds) | Admitted Patients | n=30 | Clinical Assessment | Multidisciplinary Team |  | 19 patients discharged at least one day earlier than planned | One patient was readmitted to continued treatment for the original condition. | Referral pathways with community care, nursing home beds and services were integrated in hospitals’ External Disaster Plan |  |  |
| Kelen GD, et al.^16^ | 2017 | Original Study | Retrospective Blocked Randomised | Analysis of electronic medical records | US | Simulated; not specified | N=1 Tertiary hospital (160 Beds) | Pediatric admitted patients; neonatal ICU excluded | N=501 | Expert panel determined a list of pediatric critical interventions that, if not initiated or withdrawn, could result in a consequential medical event (CME). CME: unexpected death, irreversible impairment, or reduction of function occurring within 96 hours after withdrawing or failing to initiate a PCI. Patients were eligible for early discharge if they did not require a Pediatric Critical Intervention from the start of the mock disaster through the end of day 4 | - | Applied criteria proposed by Kelen GD, et al.(2015) | 10.8% eligible for immediate low-risk reverse triage 13.2% for discharge by 96 hours. | - | Discharged home | More than half of the potential effect of reverse triage appears to be dependent on patients in the psychiatry unit (interchangeable to receive general medical patients?) | (I)Ethical consideration regarding the utilitarian approach to resource allocation for children; (II) Transfer-of-care protocols should be outlined in the institution’s disaster surge plan (III) No risk stratification tool should be a substitute for clinical evaluation (IV) Large volumes of pediatric patients discharged early to the community during disasters could challenge pediatricians owing to the close follow-up likely to be required |
| Pollaris G, et al.^22^ | 2018 | Original study | Prospective, quantitative | Expert panel + randomised controlled pilot study | Belgium | Simulated; not specified | Large tertiary hospital in Leuven, Belgium | Adult and Pediatric admitted patients; ICU and some specialties excluded | N=490 | Reverse Triage Tool Leuven (RTTL) | Multidisciplinary team (physician, nurse, social assistant) | Based on Kelen classification system (risk assessment of consequential medical events resulting from early hospital discharge, if a Critical Intervention is not needed. RTTL works by exclusion, patients excluded based on their need for a critical intervention | - | - | - | Having pre-defined criteria is a facilitator: RTTL saves time reducing the patient population to evaluate for potential early discharge to one-third and doubles the probability of selecting an actual dischargeable patient | To equip hospitals with checklist, which saves time, doubles the probability of actual discharge in disaster circumstances |
| Jacobs-Wingo JL, et al.^17^ | 2018 | Original study | Prospective, quantitative | Survey | US | Simulated; not specified | N=45 Hospitals with a total bed capacity of 19.733. N=6 (13.3%) hospitals had fewer than 200 inpatient beds, N=16 (35.6%) had between 200 and fewer than 400 inpatient beds, and N=23 (51.1 %) had 400 or more inpatient beds. | Admitted patients | - | Rapid Patient Discharge Assessment Tool (RPDA Tool) | - | The Department of Health and Mental Hygiene (DOHMH) created the Rapid Patient Discharge Tool (RPD Tool) based on activities and conditions found to contribute to the majority of discharges. Updated in RPDA Tool in 2011. | Selected N=4225 patients through the RPDA (21.4% of total bed capacity.):26.9% were already confirmed for discharge; 43.9% were round 1 discharge candidates; and 29.2% were round 2 discharge candidates. | - | Round 1: 61,9% discharged to home and 24.7% being discharged to a healthcare facility.  Round 2: 60.2% discharged to home and 27.3% discharged to a healthcare facility. | :  (I) Factors affecting discharge process: missing prescription for aftercare, missing discharge order, laboratory results, clothing not available, interpreter needed, radiology test needed, on ventilator (II) need for discharge planner (III) factors affecting continuity of care: inability to engage daily activities, home healthcare needs, functional disability, caretaker unavailable to meet postdischarge needs and (IV) transportation mode: Self, Family or friend, Ambulance, Ambulette. | Clinical staff should develop solutions to frequently occurring barriers and incorporate them into routine discharge planning |
| Esmailian M, et al.^21^ | 2018 | Original Study | Cross sectional | Survey | Iran | Simulated; not specified | Academic Hospital (800 active beds) | Admitted patients | - | Hospital instructions for decision making based on Handbooks of Emergency Medicine on the ten most common diseases leading to hospitalization in each ward of the hospital. | A Preselected in-charge person of each department | An internal expert panel developed the guidelines for disposition of the patients hospitalised | N=108 (20%) hospitalised patients suitable for discharge | - | - | Patients’ clinical profile:orthopedics, urology, pediatric surgery, plastic surgery, male surgical ward, neurosurgery, and ENT wards have the greatest potential for RT | (I) RT is unlikely to represent an effective solution to surge outside of a disaster setting because of its requirement for centralized decision making. (II) Since in different studies the risk stratification for early discharge is not the same, RT gives different results for surge capacity (III) Referral centers could have a greater percentage of critically ill patients compared to smaller hospitals where RT may result more effective. |
| Bird R, et al.^20^ | 2020 | Original study | Prospective cross-sectional | Pilot study | UK | Simulation of mustard gas attack | Pediatric major trauma center (68 ward beds; 5 PICU) | Pediatric admitted patients | N=20 Simulated patients | Free clinician-decision but largely included: (I) patients awaiting inpatient tests that could be performed as outpatients, (II) patients whose elective procedures were cancelled (III) Patients that no longer require intravenous medication | Consultant physician or senior Nurse | Internal major incident discharge protocol developed | 42% | 3 of 29 patients; 7 days Follow Up | Discharge Home | (I) Parent physically present; weaning plan for inhalers;  discharge summaries and prescriptions.  (II) Handwritten summaries took 13.3% of the time that electronic summaries took (III) discharge lounge | (I)) rapid discharge system where paperwork is minimized (II) discharge system based on different tier (III) Contingency plans in place to help source and space allocations (IV) Healthcare workers (divided in admission and discharge teams) available to manage high number of admission and discharge x |
| Burgess MC, et al.^18^ | 2021 | Original Study | Prospective, quantitative | Survey | US | Simulated; not specified | Academic Pediatric Hospital (145 beds) | Pediatric admitted patients |  | Emergency Discharge Assessment Tool | patients identified through unit-level case managers, nurse and physician meetings | The tool was developed a priori, the categories of barriers to discharge were developed empirically through conversations between the study team, nursing, and case management staff. | A mean of 25 patients (22% of total bed capacity) is the expected discharge per day. In addition, an average of 14 (13%) patients per day could be discharged to an alternative care facility or home if specific additional services could be provided. | - | Home; alternative care facility or home with specific additional services | Barrier to discharge categorised as those that could or could not be reasonably addressed outside of a traditional inpatient hospital setting.  In the first group are considered:  (I) safe discharge plans (for example equipment and/or medication management training) (II) intermittent monitoring by family member or outpatient support staff , (III) intravenous medications that are traditionally administered outside the hospital or could be changed to enteral formulation (IV) intermittent labs and/or increased oral intake | (I)Developing safe discharge plans, (II)providing some care not requiring nursing expertise, (III)providing advanced care by a nurse or other licensed medical professional (the largest gain would be a discharge area to continue basic care while training and discharge plan are completed) (IV) Development of pediatric surge plan involving resources generally not used for inpatient pediatric care(e.g.: allocation adults’ resources for older and lesser severe children) |
