# Supplementary material for: Primary Health Care and Disasters: Applying a “Whole-of-Health System” Approach through Reverse Triage in Mass-Casualty Management
Source: Prehosp Disaster Med. 2023 Sep 1;38(5):654–9. doi: 10.1017/S1049023X23006246 (PMC10548020; doi:10.1017/S1049023X23006246)
Supplement: Supplementary file 1 [file S1049023X23006246sup.zip › S1049023X23006246sup003.docx]

**File S2***:* Search Queries for PubMed, Web Of Science and Scopus

| PubMed | ("Disasters"[MeSH Terms] OR "Crowding"[MeSH Terms]) AND ("Surge Capacity"[MeSH Terms] OR "Patient Discharge"[MeSH Terms] OR "patient relocation"[Title/Abstract] OR "low risk"[Title/Abstract] OR inpatient[Title/Abstract] OR "primary healthcare"[Title/Abstract] OR "primary care"[Title/Abstract] OR ("rever*"[All Fields] AND ("triage"[MeSH Terms] OR "triag*"[All Fields]))) |
| --- | --- |
| Web Of Science | ALL=((“mass casualty” OR “crowding” OR "disaster") AND (“reverse triage” OR “early discharge” OR “ inpatients” OR “surge capacity” OR "patient relocation" OR “primary healthcare” OR “primary care”OR "low risk")) |
| SCOPUS | TITLE-ABS-KEY ( disaster OR "mass casualty" OR crowding ) AND TITLE-ABS-KEY ( "reverse triage" OR "surge capacity" OR "early discharge" OR "patient relocation" OR inpatient OR "low risk" or “primary healthcare” or “primary care” AND ( LIMIT-TO ( SUBJAREA , "MEDI" ) OR LIMIT-TO ( SUBJAREA , "NURS" ) ) AND ( LIMIT-TO ( LANGUAGE , "English" )) |
